# Are commonly used lab‐based measures of food value and choice predictive of self‐reported real‐world snacking? An ecological momentary assessment study

**DOI:** 10.1111/bjhp.12622

**Published:** 2022-08-24

**Authors:** Sarah Masterton, Charlotte A. Hardman, Emma Boyland, Eric Robinson, Harriet E. Makin, Andrew Jones

**Affiliations:** ^1^ Department of Psychology University of Liverpool Liverpool UK

**Keywords:** experimental medicine, food choice, food intake, implicit associations, snacking

## Abstract

**Objectives:**

While the assessment of actual food intake is essential in the evaluation of behaviour change interventions for weight‐loss, it may not always be feasible to collect this information within traditional experimental paradigms. For this reason, measures of food preference (such as measures of food value and choice) are often used as more accessible alternatives. However, the predictive validity of these measures (in relation to subsequent food consumption) has not yet been studied. Our aim was to investigate the extent to which three commonly used measures of preference for snack foods (explicit food value, unhealthy food choice and implicit preference) predicted self‐reported real‐world snacking occasions.

**Design:**

Ecological Momentary Assessment (EMA) design.

**Method:**

Over a seven‐day study period, participants (*N* = 49) completed three daily assessments where they reported their healthy and unhealthy snack food consumption and completed the three measures of preference (explicit food value, unhealthy food choice and implicit preference).

**Results:**

Our findings demonstrated some weak evidence that unhealthy Visual Analogue Scale scores predicted between‐subject increases in unhealthy snacking frequency (OR = 1.018 [1.006, 1.030], *p* = .002). No other preference measures significantly predicted self‐reported healthy or unhealthy snacking occasions (*p*s > .05).

**Conclusions:**

These findings raise questions in relation to the association between measures of preference and self‐reported real‐world snack food consumption. Future research should further evaluate the predictive and construct validity of these measures in relation to food behaviours and explore the development of alternative assessment methods within eating behaviour research.

**Statement of contribution**

***What is already known about this subject?***

- While much intervention work attempts to target food consumption behaviours, outside of controlled laboratory conditions it can be difficult to measure eating behaviour objectively.
- Alternative measures (such as food value, preference and choice) are often used to evaluate intervention effectiveness, however, the extent to which these measures are related to real world food consumption is unknown.

***What does this study add?***

- This study was the first to directly investigate the association between measures of food value and choice and self‐reported real‐world food intake
- Our findings revealed that there was limited evidence to support associations between measures of preference and healthy/unhealthy food consumption
- This raises questions in relation to the use of these strategies to evaluate intervention efficacy

## INTRODUCTION

The development of interventions that reduce motivation to consume unhealthy foods are essential to reducing the prevalence of overweight and obesity in society, and the associated burden of disease (GBD 2019 Risk Factors Collaborators, 2020; Scarborough et al., 2011). Theory and intervention development often requires *proof‐of‐concept* testing in the laboratory, under Experimental Medicine Framework approaches (Field et al., 2020, Sheeran et al., 2019), by which candidate variables of interest are assessed and/or modified before participants are given fixed or ad‐libitum meals. Such lab‐based measures of eating behaviour allow for precise measurement under controlled and manipulable conditions (Blundell et al., 2010). However, this increased control comes at a cost. Strategies utilized in the laboratory (such as the presence of an observer during test meals) can heighten participants' awareness that their consumption is being monitored (Robinson et al., 2015), which may lead to smaller effects in the laboratory than the real world (Gough et al., 2021). Unfortunately, long term, direct measurement of eating behaviour is difficult outside of controlled, laboratory settings.

Given the difficulty in unobtrusively measuring energy intake, researchers often turn to alternative measures of eating‐behaviours, including the measurement of self‐reported *current* food value and motivation to eat. Within experimental medicine approaches, these measures have become critical in the evaluation and development of theoretical models and interventions to identify paradigms with the greatest potential for real life behavioural change, and to isolate possible mechanisms of action (Field et al., 2020). While various measures of value and preference are utilized throughout the literature, some of the most commonly used measures include hedonic food value ratings (where participants are presented with food images and asked to rate images on a Visual Analogue Scale for valence [e.g. Burger et al., 2011; Chen et al., 2018; Lawrence et al., 2015]), explicit (or forced) choice tasks (where participants are presented with food images and asked to select the item(s) that they would like to consume (e.g. Charbonnier et al., 2015; Hollands & Marteau, 2016; Kakoschke et al., 2018)) and implicit association tasks (IAT: where response latencies to categorization tasks are used to infer preferences for healthy or unhealthy items [e.g. Greenwald et al., 1998; Houben et al., 2012; Nederkoorn et al., 2010]). As such, intervention successes are often evaluated in terms of reductions in unhealthy food value or increases in healthier explicit choices (e.g. Chen et al., 2018, 2019; Hensels & Baines, 2016; Hollands et al., 2011; Hollands & Marteau, 2016; Kakoschke et al., 2018; Miguet et al., 2020; Veling et al., 2013).

Despite their widespread use, these measures have been criticized for a lack of construct validity. Klein and Hilbig (2019) suggest that the hypothetical nature of preference and choice tasks (in which there are no real‐life consequences for the participant) may bias behavioural outcomes. While many measures rely on single assessments of preference or choice, weight change (and related food intake) is the result of sustained behavioural change, and evaluating intervention efficacy (and predicting longer term behavioural change) from a single measurement has implications for the translation of results to real world contexts (given the variability in food selection and consumption over time within individuals) (Loyka et al., 2020). To date, the predictive validity of preference and choice measures (in relation to self‐reported snack food consumption) has yet to be formally investigated.

Ecological momentary assessment (EMA) techniques are well‐placed for examining the predictive validity of these measures. EMA designs allow for repeated measurement of behaviour *within* individuals, in their everyday life. They overcome many of the limitations of lab‐based research. For example, traditional retrospective recall methods (such as 24‐h recall or food frequency questionnaires) can lead to biased estimates of food consumption (Hebert et al., 1997, 2008; Schoch & Raynor, 2012; Shim et al., 2014). Additionally, allowing participants to go about their daily lives without direct observation means that eating behaviour is less likely to be supressed (Gough et al., 2021). As such, EMA designs allow researchers to measure food behaviours ‘in the moment’, which is thought to increase reporting accuracy, reduce participant burden and increase the ecological validity of outcome data (Maugeri & Barchitta, 2019). Traditional laboratory measures (such as the IAT) have successfully been applied to EMA contexts, with previous work investigating smoking behaviours discovering that lab‐assessed IAT preferences for smoking stimuli were also observed during EMA IAT assessments for participants who smoked (compared to non‐smoking participants) (Waters et al., 2010).

Although EMA studies have been used to measure food‐related behaviours in real‐world contexts (see Elliston et al., 2017; Powell et al., 2017; Zenk et al., 2014), no study to date has investigated the associations between measures of food value/choice and self‐reported real world snack food consumption (i.e. are experimental measures of food preference and choice associated with real world eating behaviour?). Here, we chose to examine snack food consumption rather than typical meals, as many laboratory eating assessment paradigms (e.g. ad‐libitum: Robinson et al., 2017) focus on snack‐foods, and snacking is thought to contribute to increased overall daily energy intake (Mattes, 2018). While reducing energy intake is a key aim of many studies within the research area, highly controlled laboratory experiments monitoring longitudinal food consumption in response to an intervention may not be practical (or applicable to real world contexts) (de Castro, 2000; Gibbons et al., 2014; Gough et al., 2021). It is therefore important to evaluate the extent to which easily administered measures of value and choice are related to reports of real‐world snack food consumption (Field et al., 2020).

Therefore, the aim of this study was to investigate whether three commonly used measures of food value and choice (implicit preferences, unhealthy food choices, explicit food value) predicted self‐reported snacking behaviour across a 7‐day period. We hypothesized that the measures of preference, choice and value would significantly predict healthy and unhealthy snacking occasions within the same assessment window over a 7‐day study period. The study was pre‐registered on OSF: https://osf.io/tswb2/. We also investigated the associations between implicit and explicit proxy measures in exploratory analyses.

## METHOD

### Participants

In line with our pre‐registered sampling strategy, we recruited 50 participants (based on recommendations for multi‐level modelling approaches [Maas & Hox, 2005]) and required a minimum of 50% assessment compliance for inclusion within the sample. Forty‐nine participants completed at least 11 (50%) study period assessments in addition to baseline measurements and were retained. Participants were aged between 18 and 51 years (*M* = 26.82, *SD* ± 9.58), with 24 males (*M* = 32.92 ± 8.62) and 25 females (*M* = 20.96 ± 6.27), with an average Body Mass Index (BMI) of 23.38 kg/m^2^ (± 3.30). To be eligible for participation, participants were required to be aged over 18, self‐report no history of eating disorders, follow an omnivorous or vegetarian diet, have access to a smartphone with a camera and not be attempting to lose weight (or have recently dieted). Participants were recruited through online advertisements and the wider student and staff community at the University of Liverpool. Participants recruited through online advertisements received a shopping voucher, with the value dependent upon the number of EMA assessments completed (>70% completed = £20 voucher, 50%–69% completed = £10 voucher). University of Liverpool students could participate for course credit, where a similar compensation structure was used (>70% completed = 10 points, 50%–69% completed = 5 points). The study was approved by the Local Research Ethics Committee (approval code: 7617). Testing took place during the covid‐19 pandemic (November–December 2020).

### EMA measures

#### Implicit preference

The Brief Implicit Association Task (BIAT, Sriram & Greenwald, 2009) was used to measure implicit preference for healthy (e.g. banana, carrots) and unhealthy (e.g. biscuits, cheese) food items. Participants completed 4 blocks consisting of 20 trials (total 80 trials) in addition to two short unrecorded practice blocks (14 trials each). During each block, participants were asked to sort words (positive and negative) and images (healthy and unhealthy food items) into either a combined category (e.g. healthy foods and positive words) or an ‘anything else’ category. Participants were asked to respond using the on‐screen keyboard, using the ‘I’ (if the item belonged to the combined category) and ‘E’ (if the item belonged to the anything else category) buttons. The combined category labels were either healthy‐positive (i.e. healthy foods and positive words) or unhealthy‐positive (i.e. unhealthy foods and positive words) combinations, with response latencies recorded for each trial. Participants completed two blocks of each type, the order of which was counterbalanced dependent upon session number. In line with recommendations (Nosek et al., 2014), the *D* algorithm for BIAT was used to calculate implicit preference scores, which included the removal of trials >10,000 ms in length in addition to the removal of assessments where more than 10% of trials were completed in less than 300 ms (*N* = 55 assessments total, 6% of completed assessments). Positive scores indicated a preference towards healthy food items, and negative scores indicated a preference towards unhealthy food items.

#### Explicit choice

Explicit preference for healthy and unhealthy food items was assessed through the use of a forced choice task, where participants were required to select 2 out of 8 snack food images (4 healthy options, 4 unhealthy options) that represented the foods that they would most like to consume at that moment (e.g. Hollands & Marteau, 2016). The images presented consisted of equal numbers of sweet (e.g. ice cream, pineapple) and savoury (e.g. pretzels, celery sticks) items. To prevent fatigue from repeated assessments, set blocks of images were randomly presented to participants at each assessment (ensuring that identical images were not presented in subsequent assessments and images reflected equal numbers of healthy/unhealthy sweet/savoury options). Healthy food choices were scored as +1 and unhealthy food choices were scored as 0, which when combined resulted in an explicit preference score ranging from 0 (two unhealthy selections) to 2 (two healthy selections).

#### Food value

Participants were presented with 10 images of snack food items (5 unhealthy, 5 healthy) and asked to rate each item on a Visual Analogue Scale (VAS) ranging from −100 (not at all appealing) to +100 (extremely appealing) to assess image appeal (‘How appealing do you find this image’) (e.g. Burger et al., 2011; Masterton et al., 2021). To avoid habituation, the 5 images presented for each category during the task were randomly selected from a possible 12 snack food items. Mean appeal scores were calculated at each assessment for healthy and unhealthy snack food items. Ten images were used within each assessment to reduce assessment duration and participant burden.

#### Snack food recall

At each assessment, participants were provided with several free recall boxes and asked to report any healthy and unhealthy snack food items (defined as any food item not consumed as part of a main meal [Hess et al., 2016]) that they had consumed since the last assessment (‘Please list all healthy and unhealthy snack food items consumed since the last assessment. Please be as specific as possible (i.e. 30 g cashew nuts). Snack foods are classified as items consumed outside of a main meal’). Participants were asked to provide as much detail as possible (in relation to serving size/amount consumed and brand) for consumed foods, and were also asked to take photographs of snack food packaging (and servings) prior to consumption and send them to the research team. Participants were prompted to upload images at least once per day, but could upload images at any point throughout the study period. Although only the free text recall was compulsory, previous work has demonstrated that the use of images in dietary assessments supports participant recall and increases reporting accuracy (Zhao et al., 2021). The use of food images and free text recall also supported the research team in the extraction of accurate nutritional information for specific products, and identification of portion sizes (where this information was not provided by participants) (see König et al., 2021). A combined time (free text recall) and event (image upload)‐based approach increases the accuracy and ecological validity of EMA assessments, as limitations associated with solely event‐based approaches (i.e. inability to identify occasions where snacking *did not* take place) are eliminated (Maugeri & Barchitta, 2019). Therefore, while time‐based assessments were used to measure snack food consumption, this data were validated by additional information provided through event‐based assessments, improving data quality and accuracy.

The UK Nutrient Profiling Model 2004/5 (UKNPM) was used to individually profile each food item consumed by participants as ‘healthy’ or ‘less healthy’ (Department of Health, 2011). The UKNPM categorizes food items based on the healthy (fibre; protein; fruit, nuts and vegetables) and unhealthy (saturated fat; sugar; salt) components of the product (per 100 g) in addition to the amount of energy provided by the product (kJ). A score of 4 or above indicated that the product was a ‘less healthy’ snack food item (referred to as unhealthy onwards), with foods scoring 3 and below categorized as healthy. A randomly selected sample (20%) of food scores were also independently profiled by a second researcher, with an excellent agreement rate of 95% (note: scoring discrepancies would not have resulted in any changes to food categorization [healthy/unhealthy] and were resolved within the research team).

Where brand information was available (through participant descriptions and/or uploaded images), nutritional (and portion size) information was obtained through either the manufacturers website or from the Tesco UK website (largest UK supermarket chain). Where specific brand or product information was not available, information was extracted from an equivalent Tesco ‘own brand’ product for categorization and portion size information. Across all participants, 282 unique food items were profiled, with 50 categorized as ‘healthy’ and 232 as ‘unhealthy’.

### Procedure

Participants who responded to study advertisements were provided with an information sheet (via email) providing key study details including exclusion criteria, type of tasks and measures, study duration and minimum participation thresholds. Eligible participants were then sent a URL link to the baseline assessment and prompted to install the Inquisit 6 (Millisecond Software, SA) application on their smartphone, where all assessments related to the study were completed. The baseline assessment included demographic measurements (age, sex, height and weight), the creation of a unique ID number (for future correspondence) in addition to a familiarization session (including implicit food preference, food value, explicit choice). Self‐reported height and weight information was used to calculate BMI (weight (kg)/height (m^2^)). After completion of the baseline assessment, participants were sent further documentation in relation to accurately recording and reporting food consumption and were asked to contact the researcher should any issues arise with the application or completion of measures. We chose to recruit and conduct all testing online as completely online EMA studies have similar levels of compliance and data‐quality to in‐person recruitment (Carr et al., 2020).

Starting the day after the initial baseline assessment, participants were emailed a URL link to the Inquisit application three times per day at fixed intervals (12, 4 and 8 PM) for 7 consecutive days. Each assessment began with the snack food recall, followed by the measures of preference, choice and value (counterbalanced). A full list of food items (and example images) used within preference and value measures can be found at https://osf.io/tswb2/, and all images used within the study were of unbranded snack food items presented on a plain white background to avoid the potential influence of specific brand/flavour preferences. Participants were instructed to not backdate missed assessments, and where multiple assessments were completed within the same time period, data from the first valid assessment completed within that period were retained for analysis. After the 7‐day study period, participants were contacted by email, thanked for their participation and fully debriefed and reimbursed (where appropriate).

### Data reduction and analyses

We conducted multilevel logistic regressions using the ‘glmer’ function from the ‘lme4’ package in R (v1.1–27.1; Bates et al., 2015). Our predictor variables included IAT D′ score, explicit food choices and explicit value ratings of healthy and unhealthy food items. Our primary outcome variables were healthy and unhealthy snacking occasions within each assessment period (as reported by participants since their last assessment). These variables were lagged to ensure the predictor and consumption variables reflected the same assessment period(s). We also conducted exploratory analyses using the reported number of portions of unhealthy food consumed since the last assessment. In each model we also examined age, sex and BMI as predictors. Assessment‐level predictor variables were centred against the participant average (Paccagnella, 2006), to examine within‐participant variance. To disaggregate between‐participant variance the participant average was centred against the sample average (Curran & Bauer, 2011; Wang & Maxwell, 2015). Given studies often observe a reduction in compliance over time in EMA designs (see Jones et al., 2018, 2020), we also included session number as a predictor (1–21) to reduce any confounding.

To examine whether a multilevel model (with a random intercept of participant, and no predictors) was a better fit than a single level model (with no random intercept of participant, and no predictors) we examined whether there was a reduction in the AIC values for each (smaller AIC values are indicative of better fitting models, using the same data set). Here, we used the AIC change of >10 as indicative of substantial support for a multilevel model (Burnham & Anderson, 2004). Multicollinearity was assessed via Variance Inflation Factors, using the ‘performance’ package. To assess between participant associations, we computed total unhealthy and unhealthy snacking occasions per participant, and used assessment‐level averages of IAT D′ score, explicit food choices and explicit value ratings of healthy and unhealthy food items as predictors in standard regression models. Aggregating assessment level EMA data can lead to more reliable person‐level indices (Shiffman et al., 2008).

For compliance analyses, participants were deemed to have complied with the session if they had provided information on snacking behaviour on the assessment. Compliance was binary coded (0 = non‐compliance, 1 = compliance) for each assessment. We conducted a generalized linear mixed model to examine if compliance was predicted by demographic variables (age, sex, BMI), or assessment number/day of assessment (data and analysis scripts are online https://osf.io/tswb2).

## RESULTS

### Descriptive statistics for assessment‐level and outcome variables

Breakdown of assessment‐level variables are shown in Table 1. Intraclass correlation coefficients demonstrate significant within‐person variability across all assessment‐level predictors. Breakdown of assessment level‐variables by assessment day (1–7) is shown in Table S1.

**TABLE 1:** Mean values (±*SD*) of assessment‐level variables (overall and split by session number over 7‐day assessment period)

	Mean overall	Time 1 (12 PM)	Time 2 (4 PM)	Time 3 (8 PM)	ICC
Food preference					
IAT D′	.37 (.40)	.42 (.40)	.38 (.39)	.33 (.40)	.335
Explicit choice	.90 (.70)	.97 (.70)	.87 (.72)	.85 (.68)	.268
Food value					
Unhealthy food VAS	1.23 (40.29)	−.79 (41.47)	3.79 (38.86)	.76 (40.45)	.685
Healthy food VAS	9.71 (33.45)	12.81 (32.34)	10.81 (32.59)	5.50 (35.05)	.595

*Note*: IAT D′ scores range between −2 (strong preference for unhealthy foods) and +2 (strong preference for healthy foods). Explicit choice scores range between 0 (2 unhealthy choices) and +2 (2 healthy choices). Food value scores range from −100 (not at all appealing) to +100 (extremely appealing).

Abbreviation: ICC, intraclass correlation coefficient (the association between observations within individuals).

On average participants reported consuming 5.06 (± 6.12: Range 0–26.00) healthy snack portions and 12.28 (± 7.95: Range 0–35.33) unhealthy snack portions over the 7‐day period. There was a significant difference between the two (t(48) = −5.98, *p* < .001, *d* = 1.01 [95% CI: .59 to 1.42]), but also a positive correlation (*r* = .300 [95% CI .020 to .534], *p* = .037), see Figure S1.

### Compliance

Out of 1029 possible assessments (49 participants x 21 assessments), participants completed 834 (81.0%), which is comparable to previous studies (e.g. Powell et al., 2017). On average, participants completed 17.02 assessments (*SD* = 3.55, range: 11–21).

Age (OR = 1.017 [95% CI: .972 to 1.065], *z* = .748, *p* = .454), sex (OR = .742 [95% CI: .299 to 1.751], *z* = .715, *p* = .474) and BMI (OR = 1.001 [95% CI: .874 to 1.145], *z* = .011, *p* = .991) were not significant predictors of compliance. However, assessment number (1–21) was (OR = .920 [95% CI: .893 to .947], *z* = 5.605, *p* < .001), whereby compliance decreased over the duration of the study. Additional confirmation of this was that assessment day (1–7) was also a significant negative predictor (OR = .777 [95% CI: .711 to .848], *z* = 5.586, *p* < .001).

### Confirmatory hypotheses

#### Predictors of ‘unhealthy’ snacking occasions within and between individuals

There were 328 unhealthy snacking occasions. The AIC for the null model was 1073.3 and the AIC for the multi‐level model was 976.3, indicating the multi‐level model was a substantially better fit of the data. The only significant predictor in the model was session number (OR = .962 [95% CI: .929, .995]), which was associated with a reduction in snacking over time (see Table 2). The model including predictors had a substantial reduction in AIC value (AIC = 760.0). There was some evidence of moderate multicollinearity (explicit choice between‐participants VIF = 5.79). Removal of this variable from the model led to unhealthy food VAS becoming a significant between‐participants predictor (OR = 1.018 [95% CI: 1.006, 1.030], *Z* = 3.091, *p* = .002) alongside session number. There was no significant improvement in AIC (761.8).

**TABLE 2:** A multilevel model predicting unhealthy snacking occasions

	Odds ratio	95% CI	*Z* stat.
Intercept	.706	.171, 2.911	
Demographics & Time			
Age	.996	.948, 1.046	−.150
BMI	1.016	.912, 1.133	.300
Sex	1.173	.482, 2.853	.352
Session number	.962	.929, .995	−2.232
Within‐subject			
D′ Score	1.355	.738, 2.488	.981
Explicit choice	.731	.530, 1.007	−1.913
Unhealthy VAS	.994	.986, 1.003	−1.207
Healthy VAS	1.002	.993, 1.011	.506
Between‐subject			
D′ score	1.008	.176, 5.770	.009
Explicit choice	8.759	.981, 78.14	1.943
Unhealthy VAS	1.003	.985, 1.022	.877
Healthy VAS	1.021	.999, 1.044	1.889

*Note*: Sex (male ref. category).

#### Predictors of ‘healthy’ snacking occasions within and between individuals

There were 160 healthy snacking occasions. The AIC for the null model was 797.7 and the AIC for the multi‐level model was 665.0 indicating the multilevel model was a better fit of the data. The only significant predictor in the model was session number (OR = .927 [95% CI: .930, .996]), which was associated with a reduction in snacking over time (see Table 3). There was some evidence of multicollinearity (explicit choice between‐participants VIF = 5.05). Removal of this variable from the model did not influence the pattern of results.

**TABLE 3:** A multilevel model predicting healthy snacking occasions

	Odds ratio	95% CI	*Z* stat.
Intercept	8.457	.050, 142.17	
Demographics & Time			
Age	.949	.872, 1.034	−1.179
BMI	.972	.811, 1.164	−.306
Sex	.341	.076, 1.518	−1.411
Session number	.927	.885, .972	−3.149
Within‐subject			
D′ Score	.619	.287, 1.333	.417
Explicit choice	1.309	.860, 1.991	1.257
Unhealthy VAS	.989	.978, 1.001	−1.765
Healthy VAS	1.004	.993, 1.015	.765
Between‐subject			
D'Score	.362	.020, 6.568	−.686
Explicit choice	3.300	.107, 101.20	.684
Unhealthy VAS	.996	.966, 1.097	−.232
Healthy VAS	1.002	.968, 1.037	.165

*Note*: Sex (male ref. category).

### Exploratory hypotheses

#### Do measures of food value predict unhealthy snack portions?

Of the 328 unhealthy snacking occasions we examined the number of portions of unhealthy snacks as an outcome. The average number of portions was 1.63 (± 1.33). There were no significant predictors (see Appendix S1 for full model reporting). We did not replicate this analysis with healthy snacks, due to the smaller number of snacking occasions.

#### Do within‐subject explicit measures of food value and choice predict implicit measures?

We examined assessment‐level associations between explicit measures of value/choice (healthy VAS scores, unhealthy VAS scores and explicit choice) on implicit value (IAT D′ score). There was a significant association between healthy VAS scores and IAT D′ (*b* = .001[95% CI: >.001 to .002], *z* = 2.028, *p* = .042), but not with unhealthy VAS scores (*b* < .000, *p* = .935) or explicit choice (*b* = .028, *p* = .153). Variance inflation factors were <1.05.

## DISCUSSION

The aim of this study was to investigate the predictive validity of commonly used measures of food value and choice (food value, explicit choice, implicit preference) in relation to self‐reported real‐world healthy and unhealthy snack food consumption. The results demonstrated that, aside from unhealthy food VAS ratings, the preference measures were not robust predictors of healthy or unhealthy snacking occasions, and they also failed to predict the number of unhealthy snack portions consumed by participants. There were also no robust significant associations between individual measures of preference and choice, with the exception of healthy food value and IAT D′ score, which may suggest that each of these measures are unlikely to relate to the same underlying construct.

Due to the extensive use of these measures throughout the literature, we predicted that the measures would be significant predictors of both healthy and unhealthy snack food consumption. However, this does not appear to be the case, as only unhealthy food VAS scores significantly predicted self‐reported consumption behaviour within the study, and only within a model in which removal of parameters influencing multi‐collinearity was undertaken (and this model was not an improved fit of the data). These findings are important as they may help to explain poor or inconsistent translations (in relation to theoretic predictions and behavioural change) between laboratory studies and clinical interventions where measures of food preference and choice have been used to evaluate outcomes: Field et al. (2020) suggest that while experiments can demonstrate causality within a controlled environment, interventions based upon these manipulations may not be feasible should outcomes not equate to desirable (and sustained) behavioural change. Significant changes to food preference and choice using measures similar to those tested in this study have been documented within several intervention studies (e.g. Chen et al., 2018, 2019; Hensels & Baines, 2016; Kakoschke et al., 2018), however, based on the present research it remains unclear whether these would translate to changes in snacking behaviour in the real‐world.

One potential reason for a lack of consilience between preference measures and actual eating behaviour may be related to the nature of choice and preference measures within appetite research: responses have no real consequences for participants (Klein & Hilbig, 2019); therefore they may not be motivated to respond in a way that reflects their true food preferences or current underlying motivation. The findings from this study raise questions in relation to the ability of food value and choice measures to predict future consumption behaviours, which has implications for the development and evaluation of current and future weight‐loss interventions.

Interestingly, the results also revealed that different preference measures did not necessarily relate to each other within individuals (the association between IAT D′ and healthy VAS scores aside). Given that these measures are hypothesized to measure the similar constructs, some level of association would be anticipated between these variables (i.e. an implicit preference for healthy foods would be associated with increased healthy food value and healthier explicit choices). This finding may help to explain some of the inconsistencies observed within previous research: while Hollands and Marteau (2016) found that exposure to negative health‐related images led to increased explicit preference for fruit (within a forced choice task), there was no significant parallel effect on implicit preferences. The lack of association between preference measures could be related to the manner in which tasks are presented: explicit choice tasks are often relatively short, and participants are able to easily control and manipulate their responses, unlike implicit preference measures, which are indirect and more complex (with the ‘desirable’ response less obvious) (Goodall, 2011).

We demonstrated that compliance with EMA assessments decreased over time, which is common within EMA studies (Jones et al., 2020; Maugeri & Barchitta, 2019). The results also revealed that both healthy and unhealthy snacking significantly decreased during the study period (despite participants not reporting attempting to lose or reduce weight before participating). While it is possible that continued self‐monitoring of behaviour reduced snack food consumption over time (e.g. Humphreys et al., 2021; Michie et al., 2009), reductions may be indicative of reduced engagement with assessments, or participants may have deliberately chosen to miss assessments/not report snacking occasions towards the end of the study (due to pressures associated with continual monitoring of food intake/study duration (Doherty et al., 2020)). As such, a potential limitation of this research is that we were not modelling *naturalistic* snacking behaviour or capturing all potential snacking outcomes. The EMA procedure we adopted is widely used, but its validity as a measure of snacking behaviour has not been tested. In addition, because snacking behaviour was self‐reported (and will therefore be prone to bias), it may be the case participants chose not to report snacking occasions in an attempt at impression management/self‐presentation (Vartanian, 2015). Therefore, future research should examine if preference measures would be more strongly associated with objectively measured snacking behaviour (such as data collected through wearable technology devices [Skinner et al., 2020]).

Whilst BMI was included within both models, it was not a significant predictor of healthy or unhealthy snack food occasions. The average participant BMI fell within the ‘healthy’ range, and while previous work has found no significant association between BMI and laboratory assessments of food consumption (Robinson et al., 2017), it is possible that individuals with overweight or obesity may exhibit specific consumption (and preference) behaviours not observed within healthy weight groups (Mattes, 2014; Rodrigues et al., 2012). As individuals with overweight and obesity are often a key target for weight reduction interventions, future research should investigate associations between preference and consumption within this specific group to identify any potential differences in predictive validity of choice and preference measures (based upon weight status). Future work could also measure additional participant level factors (such as dietary restraint and hunger) to investigate potential associations between these variables and measures of food preference/consumption.

The use of an EMA design allowed for the examination of real‐world snack food consumption and preference over a seven‐day period, however, there were limitations associated with this approach. Participants completed assessments within fixed time periods, which may have introduced issues in relation to recall accuracy (as participants would have to wait for the next assessment to report snack foods consumed irrespective of snack timing). While participants were asked to photograph consumed snack foods and upload images (to support recall between assessments), future research could explore the incorporation of event‐contingent assessments within studies, where participants initiate assessments at each consumption occasion (although this reduces reporting and can make reviewing compliance more difficult (Maugeri & Barchitta, 2019)). Additionally, while EMA allows participants to complete assessments in environments of their choice (increasing ecological validity), research demonstrates that environmental cues (such as advertisements, social cues and snack availability [Elliston et al., 2017]) are important predictors of consumption behaviours. Environmental variations between (and within) participants may have influenced (or prompted) snack choice and preference responses, and future research should attempt to further examine these factors by collecting information related to the context in which each assessment was completed. It is also possible that our between‐participant effects are underpowered, indeed *N* = 49 would only allow detection of relatively moderate associations in cross‐sectional analysis (*r*s ~ .22). However, we note that lab‐based studies have demonstrated effects greater than this for food‐liking and consumption (*r* = .27: Robinson et al., 2017) and VAS motivation measures and consumption (*r*s ~ .48: Hammond et al., 2022). Finally, it is worth noting that this study took place during the Covid‐19 pandemic, and research has demonstrated changes in snacking and unhealthy behaviours during this time (Bakaloudi et al., 2021; Robinson et al., 2021). Replication of these findings post‐pandemic is warranted.

In conclusion, using an EMA design, this study investigated the predictive validity of three commonly used measures of value and choice (food value, explicit preference, implicit preference) in relation to real‐world snack food consumption. The results demonstrated unconvincing evidence for their prediction of self‐reported healthy or unhealthy snacking occasions, or the number of unhealthy snack food portions consumed by participants. These findings raise uncertainties about the use of food value and preference measures as predictors of snack food consumption across the wider literature. However, it is possible that limitations with the EMA design (i.e. influencing naturalistic snacking, non‐reporting) may have obscured any relationships between these variables.

## AUTHOR CONTRIBUTIONS

**Sarah Masterton:** Conceptualization; formal analysis; investigation; methodology; writing – original draft; writing – review and editing. **Charlotte A. Hardman:** Conceptualization; methodology; writing – review and editing. **Emma Boyland:** Writing – review and editing. **Eric Robinson:** Writing – review and editing. **Harriet E. Makin:** Formal analysis; writing – review and editing. **Andrew Jones:** Conceptualization; formal analysis; methodology; writing – review and editing.

## CONFLICT OF INTEREST

None.

## Supporting information


Appendix S1

## Data Availability

The data that support the findings of this study are openly available through Open Science Framework at http://doi.org/10.17605/OSF.IO/TSWB2.

## References

[bjhp12622-bib-0001] Bakaloudi, D. R. , Jeyakumar, D. T. , Jayawardena, R. , & Chourdakis, M. (2021). The impact of COVID‐19 lockdown on snacking habits, fast‐food and alcohol consumption: A systematic review of the evidence. Clinical Nutrition. Advance online publication. 10.1016/j.clnu.2021.04.020 PMC8052604 34049747

[bjhp12622-bib-0002] Bates, D. , Mächler, M. , Bolker, B. , & Walker, S. (2015). Fitting linear mixed‐effects models using lme4. Journal of Statistical Software, 67(1), 1–48. 10.18637/jss.v067.i01

[bjhp12622-bib-0003] Blundell, J. , De Graaf, C. , Hulshof, T. , Jebb, S. , Livingstone, B. , Lluch, A. , Mela, D. , Salah, S. , Schuring, E. , Van Der Knaap, H. , & Westerterp, M. (2010). Appetite control: Methodological aspects of the evaluation of foods. Obesity Reviews, 11, 251–270. 10.1111/j.1467-789X.2010.00714.x 20122136 PMC3609405

[bjhp12622-bib-0004] Burger, K. S. , Cornier, M. A. , Ingebrigtsen, J. , & Johnson, S. L. (2011). Assessing food appeal and desire to eat: The effects of portion size & energy density. The International Journal of Behavioral Nutrition and Physical Activity, 8, 101. 10.1186/1479-5868-8-101 21943082 PMC3204278

[bjhp12622-bib-0005] Burnham, K. P. , & Anderson, D. R. (2004). Multimodel inference: Understanding AIC and BIC in model selection. Sociological Methods & Research, 33(2), 261–304. 10.1177/0049124104268644

[bjhp12622-bib-0006] Carr, D. J. , Adia, A. C. , Wray, T. B. , Celio, M. A. , Pérez, A. E. , & Monti, P. M. (2020). Using the internet to access key populations in ecological momentary assessment research: Comparing adherence, reactivity, and erratic responding across those enrolled remotely versus in‐person. Psychological Assessment, 32(8), 768–779. 10.1037/pas0000847 32437190 PMC8327321

[bjhp12622-bib-0007] Charbonnier, L. , van der Laan, L. N. , Viergever, M. A. , & Smeets, P. A. (2015). Functional MRI of challenging food choices: Forced choice between equally liked high‐ and low‐calorie foods in the absence of hunger. PLoS One, 10(7), e0131727. 10.1371/journal.pone.0131727 26167916 PMC4500585

[bjhp12622-bib-0008] Chen, Z. , Holland, R. W. , Quandt, J. , Dijksterhuis, A. , & Veling, H. (2019). When mere action versus inaction leads to robust preference change. Journal of Personality and Social Psychology, 117(4), 721–740. 10.1037/pspa0000158 30920280

[bjhp12622-bib-0009] Chen, Z. , Veling, H. , de Vries, S. P. , Bijvank, B. O. , Janssen, I. M. C. , Dijksterhuis, A. , & Holland, R. W. (2018). Go/no‐go training changes food evaluation in both morbidly obese and normal weight individuals. Journal of Consulting and Clinical Psychology, 86(12), 980–990. 10.1037/ccp0000320 30507224

[bjhp12622-bib-0010] Curran, P. J. , & Bauer, D. J. (2011). The disaggregation of within‐person and between‐person effects in longitudinal models of change. Annual Review of Psychology, 62, 583–619. 10.1146/annurev.psych.093008.100356 PMC3059070 19575624

[bjhp12622-bib-0011] de Castro, J. M. (2000). Eating behaviour: Lessons from the real world of humans. Nutrition, 16(10), 800–813. 10.1016/S0899-9007(00)00414-7 11054584

[bjhp12622-bib-0012] Department of Health . (2011). Nutrient profiling technical guidance. Department of Health.

[bjhp12622-bib-0013] Doherty, K. , Balaskas, A. , & Doherty, G. (2020). The design of ecological momentary assessment technologies. Interacting with Computers, 32(3), 257–258. 10.1093/iwcomp/iwaa019

[bjhp12622-bib-0014] Elliston, K. G. , Ferguson, S. G. , Schüz, N. , & Schüz, B. (2017). Situational cues and momentary food environment predict everyday eating behavior in adults with overweight and obesity. Health Psychology, 36(4), 337–345. 10.1037/hea0000439 27669177

[bjhp12622-bib-0015] Field, M. , Christiansen, P. , Hardman, C. A. , Haynes, A. , Jones, A. , Reid, A. , & Robinson, E. (2020). Translation of findings from laboratory studies of food and alcohol intake into behavior change interventions: The experimental medicine approach. Health Psychology, 40(2), 951–959. 10.1037/hea0001022 32955278

[bjhp12622-bib-0016] GBD 2019 Risk Factors Collaborators . (2020). Global burden of 87 risk factors in 204 countries and territories, 1990‐2019: A systematic analysis for the global burden of disease study 2019. Lancet, 396(10258), 1223–1249. 10.1016/S0140-6736(20)30752-2 33069327 PMC7566194

[bjhp12622-bib-0017] Gibbons, C. , Finlayson, G. , Dalton, M. , Caudwell, P. , & Blundell, J. E. (2014). Metabolic phenotyping guidelines: Studying eating behavior in humans. Journal of Endocrinology, 222, G1–G12. 10.1530/JOE-14-0020 25052364

[bjhp12622-bib-0018] Goodall, C. E. (2011). An overview of implicit measures of attitudes: Methods, mechanisms, strengths, and limitations. Communication Methods and Measures, 5(3), 203–222. 10.1080/19312458.2011.596992

[bjhp12622-bib-0019] Gough, T. , Haynes, A. , Clarke, K. , Hansell, A. , Kaimkhani, M. , Price, B. , Roberts, A. , Hardman, C. A. , & Robinson, E. (2021). Out of the lab and into the wild: The influence of portion size on food intake in laboratory vs. real‐world settings. Appetite, 162, 105160. 10.1016/j.appet.2021.105160 33556391

[bjhp12622-bib-0020] Greenwald, A. G. , McGhee, D. E. , & Schwartz, J. L. K. (1998). Measuring individual differences in implicit cognition: The implicit association test. Journal of Personality and Social Psychology, 74(6), 1464–1480. 10.1037/0022-3514.74.6.1464 9654756

[bjhp12622-bib-0021] Hammond, L. , Morello, O. , Kucab, M. , Totosy de Zepetnek, J. O. , Lee, J. J. , Doheny, T. , & Bellissimo, N. (2022). Predictive validity of image‐based motivation‐to‐eat visual analogue scales in normal weight children and adolescents aged 9–14 years. Nutrients, 14(3), 636. 10.3390/nu14030636 35276995 PMC8840208

[bjhp12622-bib-0022] Hebert, J. R. , Hurley, T. G. , Peterson, K. E. , Resnicow, K. , Thompson, F. E. , Yaroch, A. L. , Ehlers, M. , Midthune, D. , Williams, G. C. , Greene, G. W. , & Nebeling, L. (2008). Social desirability trait influences on self‐reported dietary measures among diverse participants in a multicentre multiple risk factor trial. The Journal of Nutrition, 138(1), 226S–234S. 10.1093/jn/138.1.226S 18156429

[bjhp12622-bib-0023] Hebert, J. R. , Ma, Y. , Clemow, L. , Ockene, I. S. , Saperia, G. , Stanek, E. J., III , Merriam, P. A. , & Ockene, J. K. (1997). Gender differences in social desirability and social approval bias in dietary self‐report. American Journal of Epidemiology, 146(12), 1046–1055. 10.1093/oxfordjournals.aje.a009233 9420529

[bjhp12622-bib-0024] Hensels, I. S. , & Baines, S. (2016). Changing ‘gut’ feelings about food: An evaluative conditioning effect on implicit food evaluations and food choice. Learning and Motivation, 55, 31–44. 10.1016/j.lmot.2016.05.005

[bjhp12622-bib-0025] Hess, J. M. , Jonnalagadda, S. S. , & Slavin, J. L. (2016). What is a snack, why do we snack, and how can we choose better snacks? A review of the definitions of snacking, motivations to snack, contributions to dietary intake, and recommendations for improvement. Advances in Nutrition, 7(3), 466–475. 27184274 10.3945/an.115.009571 PMC4863261

[bjhp12622-bib-0026] Hollands, G. J. , & Marteau, T. M. (2016). Pairing images of unhealthy and healthy foods with images of negative and positive health consequences: Impact on attitudes and food choice. Health Psychology, 35(8), 847–851. 10.1037/hea0000293 27505205

[bjhp12622-bib-0027] Hollands, G. J. , Prestwich, A. , & Marteau, T. M. (2011). Using aversive images to enhance healthy food choices and implicit attitudes: An experimental test of evaluative conditioning. Health Psychology, 30(2), 195–203. 10.1037/a0022261 21401253

[bjhp12622-bib-0028] Houben, K. , Havermans, R. C. , Nederkoorn, C. , & Jansen, A. (2012). Beer à no‐go: Learning to stop responding to alcohol cues reduces alcohol intake via reduced affective associations rather than increased response inhibition. Addiction, 107(7), 1280–1287. 10.1111/j.1360-0443.2012.03827.x 22296168

[bjhp12622-bib-0029] Humphreys, G. , Evans, R. , Makin, H. , Cooke, R. , & Jones, A. (2021). Identification of behavior change techniques from successful web‐based interventions targeting alcohol consumption, binge eating, and gambling: Systematic review. Journal of Medical Internet Research, 23(2), 22694. 10.2196/22694 PMC7902193 33560243

[bjhp12622-bib-0030] Jones, A. , Crawford, J. , Rose, A. , Christiansen, P. , & Cooke, R. (2020). Regret me not: Examining the relationship between alcohol consumption and regrettable experiences. Substance Use & Misuse, 55(14), 2379–2388. 10.1080/10826084.2020.1817084 32924717

[bjhp12622-bib-0031] Jones, A. , Tiplady, B. , Houben, K. , Nederkoorn, C. , & Field, M. (2018). Do daily fluctuations in inhibitory control predict alcohol consumption? An ecological momentary assessment study. Psychopharmacology, 235(5), 1487–1496. 10.1007/s00213-018-4860-5 29497782 PMC5919991

[bjhp12622-bib-0032] Kakoschke, N. , Hawker, C. , Castine, B. , de Courten, B. , & Verdejo‐Garcia, A. (2018). Smartphone‐based cognitive bias modification training improves healthy food choice in obesity: A pilot study. European Eating Disorders Review, 26(5), 526–532. 10.1002/erv.2622 30003634

[bjhp12622-bib-0033] Klein, S. A. , & Hilbig, B. E. (2019). On the lack of real consequences in consumer choice research: And its consequences. Experimental Psychology, 66(1), 68–76. 10.1027/1618-3169/a000420 30777510

[bjhp12622-bib-0034] König, L. M. , Van Emmenis, M. , Nurmi, J. , Kassavou, A. , & Sutton, S. (2021). Characteristics of smartphone‐based dietary assessment tools: A systematic review. Health Psychology Review. Advance online publication. 10.1080/17437199.2021.2016066 34875978

[bjhp12622-bib-0035] Lawrence, N. S. , O'Sullivan, K. , Parslow, D. , Javaid, M. , Adams, R. C. , Chambers, C. D. , Kos, K. , & Verbruggen, F. (2015). Training response inhibition to food is associated with weight loss and reduced energy intake. Appetite, 95, 18–28. 10.1016/j.appet.2015.06.009 PMC4596151 26122756

[bjhp12622-bib-0036] Loyka, C. M. , Ruscio, J. , Edelblum, A. B. , Hatch, L. , Wetreich, B. , & Zabel, A. (2020). Weighing people rather than food: A framework for examining external validity. Perspectives on Psychological Science, 15(2), 483–496. 10.1177/1745691619876279 31743074

[bjhp12622-bib-0037] Mattes, R. (2014). Energy intake and obesity: Ingestive frequency outweighs portion size. Physiology and Behavior, 134, 110–118. 10.1016/j.physbeh.2013.11.012 24291535

[bjhp12622-bib-0038] Mattes, R. (2018). Snacking: A cause for concern. Physiology & Behavior, 193, 279–283. 10.1016/j.physbeh.2018.02.010 29421590

[bjhp12622-bib-0039] Maas, C. J. , & Hox, J. J. (2005). Sufficient sample sizes for multilevel modeling. Methodology, 1(3), 86. 10.1027/1614-2241.1.3.86

[bjhp12622-bib-0040] Masterton, S. , Hardman, C. A. , Halford, J. C. G. , & Jones, A. (2021). Examining cognitive bias modification interventions for reducing food value and choice: Two pre‐registered, online studies. Appetite, 159, 105063. 10.1016/j.appet.2020.105063 33279528

[bjhp12622-bib-0041] Maugeri, A. , & Barchitta, M. (2019). A systematic review of ecological momentary assessment of diet: Implications and perspectives for nutritional epidemiology. Nutrients, 11, 2696. 10.3390/nu11112696 31703374 PMC6893429

[bjhp12622-bib-0042] Michie, S. , Abraham, C. , Whittington, C. , McAteer, J. , & Gupta, S. (2009). Effective techniques in healthy eating and physical activity interventions: A meta‐regression. Health Psychology, 28(6), 690–701. 10.1037/a0016136 19916637

[bjhp12622-bib-0043] Miguet, M. , Beaulieu, K. , Fillon, A. , Khammassi, M. , Masurier, J. , Lambert, C. , Duclos, M. , Boirie, Y. , Finlayson, G. , & Thivel, D. (2020). Effect of a 10‐month residential multidisciplinary weight loss intervention on food reward in adolescents with obesity. Physiology & Behavior, 223, 112996. 10.1016/j.physbeh.2020.112996 32505785

[bjhp12622-bib-0044] Nederkoorn, C. , Houben, K. , Hofmann, W. , Roefs, A. , & Jansen, A. (2010). Control yourself or just eat what you like? Weight gain over a year is predicted by an interactive effect of response inhibition and implicit preference for snack foods. Health Psychology, 29(4), 389–393. 10.1037/a0019921 20658826

[bjhp12622-bib-0045] Nosek, B. A. , Bar‐Anan, Y. , Sriram, N. , Axt, J. , & Greenwald, A. G. (2014). Understanding and using the brief implicit association test: Recommended scoring procedures. PLoS One, 9(12), e110938. 10.1371/journal.pone.0110938 25485938 PMC4259300

[bjhp12622-bib-0046] Paccagnella, O. (2006). Centering or not centering in multilevel models? The role of the group mean and the assessment of group effects. Evaluation Review, 30(1), 66–85. 10.1177/0193841X05275649 16394187

[bjhp12622-bib-0047] Powell, D. , Mcminn, D. , & Allan, J. L. (2017). Does real time variability in inhibitory control drive snacking behaviour? An intensive longitudinal study. Health Psychology, 36(4), 356–364. 10.1037/hea0000471 28192005

[bjhp12622-bib-0048] Robinson, E. , Boyland, E. , Chisholm, A. , Harrold, J. , Maloney, N. G. , Marty, L. , Mead, B. R. , Noonan, R. , & Hardman, C. A. (2021). Obesity, eating behavior and physical activity during COVID‐19 lockdown: A study of UKadults. Appetite, 156, 104853. 10.1016/j.appet.2020.104853 33038479 PMC7540284

[bjhp12622-bib-0049] Robinson, E. , Hardman, C. A. , Halford, J. C. , & Jones, A. (2015). Eating under observation: A systematic review and meta‐analysis of the effect that heightened awareness of observation has on laboratory measured energy intake. American Journal of Clinical Nutrition, 102(2), 324–337. 10.3945/ajcn.115.11119 26178730

[bjhp12622-bib-0050] Robinson, E. , Haynes, A. , Hardman, C. A. , Kemps, E. , Higgs, S. , & Jones, A. (2017). The bogus taste test: Validity as a measure of laboratory food intake. Appetite, 116, 223–221. 10.1016/j.appet.2017.05.002 28476629 PMC5504774

[bjhp12622-bib-0051] Rodrigues, A. G. M. , da Costa Proença, R. P. , Calvo, M. C. M. , & Fiates, G. M. R. (2012). Overweight/obesity is associated with food choices related to rice and beans, colors of salads, and portion size among consumers at a restaurant serving buffet‐by‐weight in Brazil. Appetite, 59(2), 305–311. 10.1016/j.appet.2012.05.018 22634196

[bjhp12622-bib-0052] Scarborough, P. , Bhatnagar, P. , Wickramasinghe, K. K. , Allender, S. , Foster, C. , & Rayner, M. (2011). The economic burden of ill health due to diet, physical inactivity, smoking, alcohol and obesity in the UK: An update to 2006‐07 NHS costs. Journal of Public Health, 33(4), 527–535. 10.1093/pubmed/fdr033 21562029

[bjhp12622-bib-0053] Schoch, A. H. , & Raynor, H. A. (2012). Social desirability, not dietary restraint, is related to accuracy of reported dietary intake of a laboratory meal in females during a 24‐hour recall. Eating Behaviors, 13(1), 78–81. 10.1016/j.eatbeh.2011.11.010 22177404

[bjhp12622-bib-0054] Sheeran, P. , Klein, W. M. , & Rothman, A. J. (2017). Health behavior change: Moving from observation to intervention. Annual Review of Psychology, 68, 573–600. 10.1146/annurev-psych-010416-044007 27618942

[bjhp12622-bib-0055] Shiffman, S. , Stone, A. A. , & Hufford, M. R. (2008). Ecological momentary assessment. Annual Review of Clinical Psychology, 4, 1–32. 10.1146/annurev.clinpsy.3.022806.091415 18509902

[bjhp12622-bib-0056] Shim, J. S. , Oh, K. , & Kim, H. C. (2014). Dietary assessment methods in epidemiologic studies. Epidemiology and Health, 36, e2014009. 10.4178/epih/e2014009 25078382 PMC4154347

[bjhp12622-bib-0057] Skinner, A. , Toumpakari, Z. , Stone, C. , & Johnson, L. (2020). Future directions for integrative objective assessment of eating using wearable sensing technology. Frontiers in Nutrition, 7, 80. 10.3389/fnut.2020.00080 32714939 PMC7343846

[bjhp12622-bib-0058] Sriram, N. , & Greenwald, A. G. (2009). The brief implicit association test. Journal of Experimental Psychology, 56(4), 283–294. 10.1027/1618-3169.56.4.283 19439401

[bjhp12622-bib-0059] Vartanian, L. R. (2015). Impression management and food intake. Current directions in research. Appetite, 86, 74–80. 10.1016/j.appet.2014.08.021 25149198

[bjhp12622-bib-0060] Veling, H. , Aarts, H. , & Stroebe, W. (2013). Stop signals decrease choices for palatable foods through decreased food evaluation. Frontiers in Psychology, 4, 875. 10.3389/fpsyg.2013.00875 24324451 PMC3840792

[bjhp12622-bib-0061] Wang, L. P. , & Maxwell, S. E. (2015). On disaggregating between‐person and within‐person effects with longitudinal data using multilevel models. Psychological Methods, 20(1), 63–83. 10.1037/met0000030 25822206

[bjhp12622-bib-0062] Waters, A. J. , Miller, E. K. , & Li, Y. (2010). Administering the implicit association test in an ecological momentary assessment study. Psychological Reports, 106(1), 31–43. 10.2466/PR0.106.1.31-43 20402424 PMC5538728

[bjhp12622-bib-0063] Zenk, S. N. , Horoi, I. , McDonald, A. , Corte, C. , Riley, B. , & Odoms‐Young, A. M. (2014). Ecological momentary assessment of environmental and personal factors and snack food intake in African American women. Appetite, 83, 333–341. 10.1016/j.appet.2014.09.008 25239402 PMC4376474

[bjhp12622-bib-0064] Zhao, X. , Xu, X. , Li, X. , He, X. , Yang, Y. , & Zhu, S. (2021). Emerging trends of technology‐based dietary assessment: A perspective study. European Journal of Clinical Nutrition, 75(4), 582–587. 10.1038/s41430-020-00779-0 33082535

