# Supplementary material for: Are commonly used lab‐based measures of food value and choice predictive of self‐reported real‐world snacking? An ecological momentary assessment study
Source: Br J Health Psychol. 2022 Aug 24;28(1):237–51. doi: 10.1111/bjhp.12622 (PMC10086796; doi:10.1111/bjhp.12622)
Supplement: Supplementary file 1 — Appendix S1 [file BJHP-28-237-s001.docx]

**XXXXXX. Are commonly used lab-based measures of food value and choice predictive of self-reported real-world snacking? An ecological momentary assessment study**

Supplementary table 1. Mean values (±SD) of assessment-level variables across 7-assessment days.

Day 1 Day 2 Day 3 Day 4 Day 5 Day 6 Day 7

Food Preference

IAT D’ 0.53 (0.38) 0.46 (0.40) 0.36 (0.41) 0.35 (0.38) 0.31 (0.40) 0.27 (0.37) 0.29 (0.39)

Explicit Choice 0.90 (0.71) 0.95 (0.67) 0.91 (0.74) 0.90 (0.69) 0.91 (0.72) 0.92 (0.67) 0.90 (0.73)

Food Value

Unhealthy food VAS 7.43 (34.27) 1.98 (40.14) 1.90 (42.80) -4.28 (40.42) -3.56(41.65) -3.07(42.25) 3.62 (40.79)

Healthy food VAS 12.06 (30.51) 12.40 (32.06) 9.80 (33.33) 8.73 (33.36) 9.34 (35.84) 10.61 (34.32) 4.26 (34.52)

*Legend: ICC = intraclass correlation coefficient (the association between observations within individuals). IAT D’ scores range between -2 (strong preference for unhealthy foods) and +2 (strong preference for healthy foods). Explicit choice scores range between 0 (2 unhealthy choices) and +2 (2 healthy choices). Food value scores range from -100 (not at all appealing) to +100 (extremely appealing).*

Supplementary figure 1. Line plot of the association between healthy and unhealthy snacks per week, by participant

*
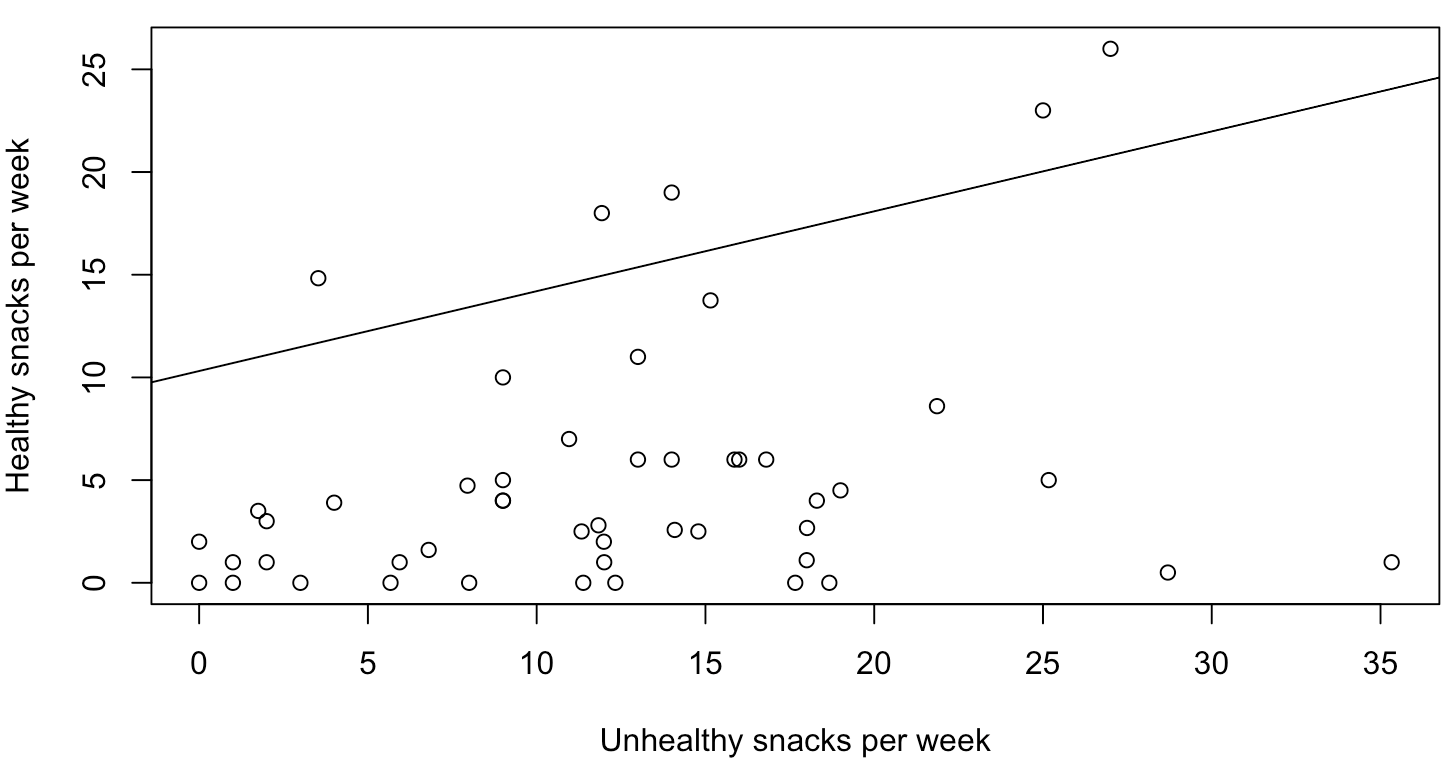
*

*Full model reporting (Unhealthy Snacking occasions)*

*healthy_binary ~ iat_center + unhealthy_val_center + healthy_val_center +*

*choice_center + BMI + Gender1M2F + Age + IAT_bs + Healthy_bs +*

*Unhealthy_bs + Explicit_bs + Session_overall + (1 | Participant_num)*

*Optimizer = ‘bobyqa’*

*AIC BIC logLik deviance df.resid*

*521.9 583.7 -247.0 493.9 596*

*Random effects variance = 2.02*

*Random effects Std. Dev = 1.48*

*Variance inflation factors*

*Low Correlation*

*Parameter VIF Increased SE*

*iat_center 1.09 1.04*

*unhealthy_val_center 1.14 1.07*

*healthy_val_center 1.07 1.03*

*choice_center 1.07 1.03*

*BMI 1.22 1.10*

*Gender1M2F 2.04 1.43*

*Age 2.30 1.52*

*IAT_bs 1.61 1.27*

*Healthy_bs 2.61 1.62*

*Unhealthy_bs 3.75 1.94*

*Session_overall 1.14 1.07*

*Moderate Correlation*

*Parameter VIF Increased SE*

*Explicit_bs 5.05 2.25*

*Full model reporting (Healthy Snacking occasions)*

*unhealthy_binary ~ iat_center + unhealthy_val_center + healthy_val_center +*

*choice_center + IAT_bs + Healthy_bs + Unhealthy_bs + Explicit_bs +*

*BMI_center + Gender1M2F + Age_center + Session_overall + (1 | Participant_num)*

*Data: EMA*

*Optimizer = ‘bobyqa’*

*AIC BIC logLik deviance df.resid*

*760.1 821.8 -366.0 732.1 596*

*Random effects variance = 0.80*

*Random Effects Std. Dev = 0.90*

*Low Correlation*

*Parameter VIF Increased SE*

*iat_center 1.09 1.04*

*unhealthy_val_center 1.11 1.05*

*healthy_val_center 1.05 1.02*

*choice_center 1.06 1.03*

*IAT_bs 1.67 1.29*

*Healthy_bs 3.16 1.78*

*Unhealthy_bs 3.88 1.97*

*BMI_center 1.20 1.09*

*Gender1M2F 1.90 1.38*

*Age_center 2.13 1.46*

*Session_overall 1.12 1.06*

*Moderate Correlation*

*Parameter VIF Increased SE*

*Explicit_bs 5.79 2.41*

*Do measures of food value predict unhealthy snack portions?*

Eleven snacking reports were outliers according to a box plot (number of portions > 3.71). As such we windzorised these to the next highest value (3.70). The two level model (occasions > participants) was a significantly better fit than a single level model (X^2^(1) = 6.98, p < .01). The variance partition coefficient was .147, indicating that 14.7% of variance was at attributable to the individual level and 85.3% at the occasion level. There was multicollinearity present within the model (Explicit choice between subjects VIF = 6.15), therefore we removed this predictor. There was also some evidence of non-linearity of residuals, therefore results should be interpreted with caution. There were no significant predictors of number of snacks consumed. See supplementary table 2.

Supplementary table 2: Multi-level model predicting the number of snacks consumed.

Coefficient 95% CI T stat.

Intercept 2.179 0.092, 3.423

*Demographics and time*

Age -.013 -.034, .008 1.103

BMI .000 -.048, .049 0.010

Sex -.116 -.493, .265 0.551

Time -.014 -.033, .005 1.453

*Within-subject*

D’ Score .107 -.424, .507 0.558

Explicit Choice -.008 -.180, .169 -0.092

Unhealthy VAS -.003 -.008, .001 1.237

Healthy VAS .003 -.004, .005 0.134

*Between-subject*

D’ Score 0.329 -.424, 1.068 0.792

Unhealthy VAS -.002 -.007, .002 1.005

Healthy VAS .001 -.007, .002 0.411

*Legend: Sex (male ref. category)*
